# Isolation and characterisation of a novel *Stenotrophomonas maltophilia* phage vB_SmaS_BCU-1 with evaluation of mammalian cell safety

**DOI:** 10.1007/s10096-025-05395-z

**Published:** 2026-01-30

**Authors:** Kashif Haq, Martin Figgitt, David Lee, Jack Spencer, Anisa Choudhry

**Affiliations:** Department of Life Sciences, School of Health Sciences, Birmingham City University, Birmingham, B15 3TN UK

**Keywords:** Stenotrophomonas maltophilia, Antibiotic resistance, Biofilm, Bacteriophage, Cell toxicity

## Abstract

*Stenotrophomonas maltophilia* (*S. maltophilia)*, is a low virulence opportunistic pathogen intrinsically resistant to a wide range of antibiotics with several virulence factors and is increasingly found in hospital and community settings, the organism is increasingly associated with biofilm infections in diabetic foot ulcers. With limited options, a novel treatment strategy is required, and the use of lytic phages presents a promising alternative. In this study, lytic phage vB_SmaS_BCU-1 was isolated from soil and propagated with a clinical *S. maltophilia* strain, isolated from a diabetic foot ulcer. Morphology characterisation and genomic analysis revealed it is a siphophage belonging to the family *Casjenviridae*, genus *Sanovirus*. Phage vB_SmaS_BCU-1 is a dsDNA virus consisting of 57,752 bp containing 75 open reading frames, with no virulence or antibiotic resistance genes found. vB_SmaS_BCU-1 was stable at a range of temperatures (4–55 °C) & pH values (4–12), has a short latent period (30 min), a large burst size (150 PFU/cell) and efficient adsorption. The phage demonstrated lysis of planktonic cells and can significantly reduce biofilm biomass. In a human fibroblast co-culture model, the phage exhibited no cytotoxicity, protected cells from bacterial-induced damage and significantly reduced the bacterial load.

**Supplementary Information:**

The online version contains supplementary material available at 10.1007/s10096-025-05395-z.

## Introduction

*Stenotrophomonas maltophilia* (*S. maltophilia*) is a Gram-Negative bacillus, found in environments associated with soil, water and plants [1]. The global incidence and prevalence of *S. maltophilia* infection over the past 15 years have increased and it has been listed as a leading drug-resistant nosocomial pathogen by the World Health Organization [2]. Regarded as an organism with low virulence, it has emerged as a highly resistant organism with a mortality rate of up to 37.5% [3], the pathogen is a causative agent for bacteraemia, pneumonia, urinary tract infections, meningitis, endocarditis [4–8], and in recent years *S. maltophilia* has been implicated in diabetic foot ulcers (DFU), diabetic foot infections (DFI), and osteomyelitis [9–12].

*S. maltophilia* is equipped with many intrinsic resistance mechanisms such as chromosomally encoded multidrug efflux pumps, the majority of these belong to the resistance-nodulation cell-division family (RND Family), Notably, major facilitator superfamily efflux pump (EFS) and ATP binding cassette family (ABC) efflux pumps have also been characterised. These pumps reduce and provide protection against diffusion of antibiotics. Furthermore, *S. maltophilia* exhibits resistance to β-lactam antibiotics via chromosomally encoded inducible β-lactamases L1 and L2. Aminoglycoside resistance is governed by modifying enzymes (aminoglycoside acetyltransferases AAC(6’)-Iz and AAC(6’)-lak), all contribute to the organism’s resistome [13–15].

*S. maltophilia* strains are known to express cell-associated virulence factors, for example the outer lipopolysaccharide layer (LPS) plays a vital role in colonization and biofilm formation, fimbriae structures such as type 1 fimbriae SMF-1 are known to adhere to epithelial cells and the type IV pilus has been implicated in correlating biofilm formation onto mammalian cells. Extra cellular virulence factors such as, proteases, phospholipases, nucleases, lipases, and haemolysins are known to contribute to cytotoxicity, in particular the protease StmPr1, StmPr2 and StmPr3 which have been associated with tissue destruction [16].

Due to the narrow spectrum of antibiotics to treat such infections, an alternative strategy is required, and phage therapy may be a promising option. Bacteriophages (phages) are biological entities that are capable of infecting and killing bacteria via the lytic replication cycle, they target bacteria through surface receptors and demonstrate selective tropism [17]. By the end of August 2025, The International Committee on Taxonomy of Viruses (ICTV) had registered thirty-one phages targeting *S. maltophilia* [18], moreover, it has been reported there may be up to 120 *S. maltophilia* phages deposited in the National Centre for Biotechnology Information (NCBI) [19].

In this current study, a new lytic phage, vB_SmaS_BCU-1 was isolated using a strain of *S. maltophilia* (SM-BCU1) cultured from a diabetic foot ulcer. Physical and genomic characterisation of the phage was undertaken; antibacterial activity and efficacy of biofilm destruction was investigated, furthermore, safety and influence of the phage was assessed using human dermal fibroblasts.

## Materials and methods

### Bacterial isolate, phage isolation and purification

A clinical *S. maltophilia* strain (SM-BCU1) isolated from a DFI (kindly, donated from Southmead Hospital, Medical Microbiology Dept. UK) was used as the host. Genomic characterisation and antibiotic-biogram can be found in Supplementary tables S1-S5 and figure S1. Available meta data can be found in the Sequence Read Archive (SRA) SUB14869520.

Soil samples (50 g) were collected in 100 ml sterile flask, elution phage buffer (150 mM NaCl, 40 mM Tris-Cl and 10 mM MgSO_4_) was added in a 1:1 ratio. Sample was manually shaken for 10 min through repetitive inversion and left overnight at 4 °C. The sample was then centrifuged at 10,000 x *g* for 15 min and supernatant passed through a 0.22 μm membrane filter to remove any unwanted bacterial debris and kept aside.

Supernatant sample was added to equal volumes of 2x LB broth supplemented with 100 mM CaCl_2_ and 150 mM MgSO_4_. 100 µl of SM-BCU1 was grown to exponential log phase and added to the supernatant. The mixture was incubated at 30 °C at 150 rpm for 18–22 h. After which chloroform at 0.1 volume of the suspension was added and incubated at room temperature for 30 min. After incubation, sample was centrifuged at 11,000 x *g* for 5 min to remove bacteria and debris. This was repeated twice more before a double-layer plaque agar assay (DLA) was performed to isolate phages [20]. A single phage was isolated and transferred to 1 ml SM buffer, vortexed thoroughly and subjected to the double-layer plaque agar assay using the original host. This particular step was repeated 5 times and lysate containing purified phage was stored at 4 °C. PEG 8000 (20%) and NaCl (2.5 M) was added to the purified lysate and incubated at 4 °C for 24 h with continuous stirring. After incubation, the lysate was centrifuged at 10,000 x *g* for 20 min to precipitate the phages. The supernatant was decanted and resultant pellet was left to soak in 500 µl of Salt-magnesium (SM) buffer (5.8 g NaCl, 2 g MgSO_4_:7H_2_O into 900 ml of distilled water, supplemented with 50 ml 1 M Tris-HCL [pH 7.4] and 5 ml 1% w/v gelatine solution) for 30 min and then resuspended into the buffer. The phage solution was stored at 4 °C until further use. Spot test and quantification of phages were performed through the double-layer agar method [21].

## Transmission electron microscopy

Morphology of phage isolated was determined by transmission electron microscopy [22] and conducted by electron microscopy suite, Open University (https://emsuite.stem.open.ac.uk/). Briefly, 10 µl of phage lysate at a concentration of 1 × 10^9^ PFU/ml was added onto a carbon coated copper grid and negatively stained with 2% uranyl acetate. Images were processed through a JEOL JEM 1400 transmission electron microscope at a voltage of 120 kV.

## Physical characterization of phage

### Multiplicity of infection determination and one-step growth curve

The multiplicity of infection (MOI) assay was performed as previously described [23], in brief, host bacteria SM-BCU1 was grown to logarithmic phase, adjusted to 10^8^ CFU/mL and mixed with phage at different MOIs (0.01,0.1, 1, 10, 100). The mixture was incubated for 4 h at 30 °C, followed by centrifugation at 10,000 rpm for 10 min. Supernatant was filtered through a 0.22 μm filter and MOI with the highest titre was determined through the double-overlay plaque assay.

The one-step growth curve assay was performed to determine latency period and burst size, 10 ml of early exponential phase (OD_600_ 0.4) culture of SM-BCU1was grown in LB media and centrifuged at 4 °C for 5 min at 5,000 x *g*. Pellet was re-suspended in 1 ml LB medium at concentration of 10^9^ CFU/ml with 1mM CaCl_2_. 100 µl of phage lysate at a multiplicity of infection (MOI) of 0.01 was added to the re-suspended pellet. The mixture was left to absorb at room temperature for 15 min. Phage-bacterial culture was then centrifuged for 5 min at 6,000 x *g* to remove unadsorbed phages. Pellet was re-suspended in 50 ml prewarmed LB broth supplemented with 1mM CaCl_2_. Sample was incubated in a shaking incubator, at 37 °C 120 rpm for 60 min. 100 µl was drawn from the sample every five minutes to determine phage titre and relative burst size through double-overlay plaque assay. Each assay was repeated three times [24].

## Adsorption assay

An adsorption assay was used to determine the adsorption of the phage by calculating the number of unadsorbed phages, as previously described [25], with slight modifications. SM-BCU1 was grown to exponential growth and bacterial population was determined through a counter chamber at a concentration of 10^9^ CFU/ml. Phage lysate was added to 100 ml of bacterial host to achieve a Multiplicity of Infection (MOI) of 0.01. The co-culture was incubated for 10 min at 37 °C and repeat sampling was performed every 5 min for up to 20 min by adding 100 µl of samples to 900 µl ice cold LB media. Samples were centrifuged 12,000 x *g* for 4 min. Supernatant was titrated through a plaque assay to determine unadsorbed phages expressed as a percentage. The adsorption rate constant was calculated as previously described [26].

### **Influence of pH and temperature on phage stability**

Stability and viability of phage was demonstrated through the effects of pH and temperature [23], by preparing known concentration of phage (10^8^ PFU^− 1^), suspending it in 2 ml sterile microcentrifuge tubes with SM buffer at various levels of pH (3–13, respectively), using 1 M hydrochloric acid (HCI) and 1 M sodium hydroxide (NaOH) to obtain the correct pH. The tubes were incubated at 37 °C for 12 h and phage titres were determined through the double- overlay assay. The effects of temperature on the phage were evaluated by incubating the phage at 4 °C, 25 °C, 37 °C, 45 °C, 55 °C and 60 °C for 60 min In both instances, Surviving phages under different pH values and temperatures was expressed as percentage of plaques obtained for treated samples compared to untreated via the double-overlay assay. All assays were repeated in triplicate.

## Host range

The Host range of the phage was performed by spot test [21], using bacterial strains available, (this included 2 environmental strains of *S. maltophilia*, 3 clinical strains of multi drug-resistant *P. aeruginosa*, *(A) baumannii* and *(B) cepacia complex*). In summary, 100 µl of log phase bacteria was cultured on to LB agar plates via the double-overlay method. 10 µl of phage at 10^8^ PFU/ml was spotted onto the plate and incubated at 30 °C for 24 h. The host range experiment was repeated for all bacteria assessed and in triplicate.

## Lysis profile assay

Lytic activity of phage and host specificity range was determined through a liquid microtitre assay [24]. Host bacterium was grown overnight in LB broth, 30 °C. Next, 500 µl of culture was added to 4.5 ml fresh LB broth and incubated for 2 h at 30 °C 120 rpm, until cell density was equivocal to exponential growth phase. 180 µl of culture was added to a sterile 96-well titre plate and mixed with 20 µl of appropriate phage, at MOI 100, 10, 1 0.1 and 0.01, untreated host culture was used as a positive control. The plate was then incubated at 37 °C with continuous shaking. Bacterial growth was measured by reading the absorbance at OD_600_) every 30 min for 10 h. Lysis curves were obtained by plotting OD against time.

### Biofilm metabolic activity

Biofilm quantification was determined through metabolic activity. The following methodology was adopted with modifications using the MBEC assay [27, 28]. Bacterial strain, SM-BCU1 was grown overnight in LB at 30 °C and 200 rpm, culture was adjusted to OD_600_ (equivalent to 1 × 10^8^ CFU/mL) and diluted to 1 × 10^7^ CFU/mL. 200 µl was dispensed into a 96-well biofilm plate. The peg lids were carefully immersed into the biofilm plate, sealed and incubated without shaking for 24 h at 30 °C. The following day, peg lids were carefully removed and washed twice in a wash microtitre plate with 200 µl 1x PBS. The biofilm plate was read at an absorbance of 0D_600_ to determine growth and sterility (data not shown). Phage was diluted to MOI 0.01, 0.1, 1 and 10 with minimal media and 200 µl of each MOI was dispensed into a 96-well test microtitre plate. Biofilm peg lids were immersed into the appropriate wells and plate was incubated for 24 h. The controls included non-treated biofilm peg, minimal media alone, media and phage with appropriate MOIs. After 4- and 8-hour treatments, peg-lids were carefully removed and washed twice as before in 1x PBS. AlamarBlue was used as a resazurin indicator and diluted to 10% of total well volume used, in minimal medium. In a separate 96-well plate, 150 µl of the diluted indicator solution was dispensed into all wells and the challenged biofilm peg-lid was immersed and sealed with parafilm. The Plate was incubated for 60 min at 37 °C and absorbance was read at 570 nm and 600 nm using a spectrophotometer. Percentage of growth inhibited was calculated with the manufacturer’s formulae, which can be found in supplementary data, figure S2 [29]. The Assay was repeated in triplicate.

Quantification of bacteria within the biofilm was determined by scraping the peg lids and dispensing contents into a sterile 1.5 ml microcentrifuge tube containing 1 ml 1% PBS. The tube was centrifuged at 10,000 x *g* for 5 min and Supernatant was removed. Pellet was washed 3 times in 1% PBS and left to air dry for 10 min. It was re-suspended in 100 µl 1% PBS, serial diluted and plated on LB agar. Results were expressed as Log_10_ density [30]. Formulae can be found in the supplementary data, figure S3.

### Phage toxicity to fibroblasts

Cytotoxicity of phage towards fibroblasts was based on ISO 10993-12 standard [31], with modifications. Human Dermal Fibroblasts were grown overnight in a 96-well tissue culture plate at 37 °C with 95% air and 5% CO_2_ in Dulbecco’s Modified Eagle Medium (DMEM) with 10% foetal bovine serum. Cell density was 1 × 10^4^ cells/ml and total volume per well was 150 µl. After incubation, media was decanted, and phage stock was diluted in Eagle’s Minimum Essential Medium (EMEM) with 10% FBS, to achieve concentrations of 10^5^ to 10^9^ PFU/ml. 100 µl of each dilution was added to the cells, 100 µl Triton-X was used as a positive control and wells with PBS 1X was considered as a negative control. The plate was incubated for 2, 4 and 8 h at 37 °C with 95% air and 5% CO_2_. After incubation, media was decanted and 100 µl non supplemented EMEM with 10% AlamarBlue was dispensed into the cells. Plate was incubated as before for 90 min; Absorbance was read at 560 nm and 605 nm. Cytotoxicity percentage differences between treated and control cells were calculated using the manufacturer’s recommended formulae (AlamarBlue Protocols | Bio-Rad).

### Phage activity within fibroblast cell membranes

Concentrations of phage ranging from 10^5^ to 10^9^ PFU/ml and their disruption of cell membranes within fibroblasts, was further investigated using a commercial Lactate dehydrogenase (LDH) assay. The Fibroblasts were grown and treated with the stated phage concentrations as mentioned in the above method. LDH release was measured via the manufacturer’s protocol (CytoTox 96^®^ Promega) with minor modifications. Sixty minutes before the end of the penultimate incubation period, 10 µl of 10X lysis buffer, acting as a positive control was added to the appropriate wells. After incubation, 50 µl of medium from all wells were transferred into a sterile 96-well microtitre plate and 50 µl of LDH substrate mix was added to each well. The Plate was incubated for 30 min in the dark at room temperature. Following incubation, 50 µl of stop solution was added and absorbance read at 490 nm, and results were expressed as percentage of LDH released [32].

### Phage activity against host bacteria within fibroblasts

To determine phage activity against infected fibroblast cells, an overnight culture of SM-BCU1 was grown to exponential phase at 30 °C, 150 rpm. Sample was centrifuged for 90 s at 10,000 x *g*, 4 °C. Supernatant was discarded and the pellet was washed twice with PBS, to remove any possible bacterial metabolites and excess media, it was then resuspended in DMEM and used immediately to inoculate cell line.

100 µl of bacterial suspension at 1 × 10^6^ CFU/ml was added and plate was incubated for 2 h. Bacteriophage at 10^7^ PFU/mL was added and plate was incubated for 2- 4-, and 8-hours. At each stoppage, 50 µl of the medium was removed, serially diluted in distilled water and 50 µl of each dilution was inoculated onto a LB agar plate. The plates were incubated for 24 h at 37 °C, the following day viable bacterial concentration was calculated using the CFU method [32, 33].

### Statistical analysis

All experiments were performed in triplicate. Results were expressed as replicate means *±* SD and differences evaluated with One-way ANOVA and Tukey-Kramer test, when required on Excel XLSTAT *P*-value < 0.05 were considered statistically significant.

### DNA extraction of bacteriophage

#### Removal of bacterial DNA and RNA

Residual bacterial DNA and RNA in phage lysate at PFU/ml of 10^9^ was treated by adding 100 µl of DNase I 10x buffer, 1 µl DNase I (1U/µl) and 1 µl RNase A (10 mg/ml) to 900 µl filter-sterilized lysate. The lysate was then incubated at 37 °C for 90 min. DNase I and RNase A activity was inhibited by adding 20 µl 0.5 M EDTA. Phage protein capsid was digested by adding 1.25 µl Proteinase K (20 mg/ml) and incubated for another 90 min at 56 °C. DNA was extracted using the NORGEN BIOTEK phage DNA isolation kit and its protocol. Qubit was used, according to the manufacturer’s instructions to quantify the concentration and quality of DNA and Nano Drop were used to quantify DNA.

### Genomic sequencing method

Samples were sequenced by microbesNG (https://microbesng.com*)*, genomic DNA libraries were prepared via the Nextera XT Library Prep Kit, following the manufacturer’s protocol, Library preparation and DNA quantification was performed on the Hamilton MicrolaB STAR automated handling system. The libraries were sequenced on Illumina NovaSeq 6000 using a 2 × 250 bp paired end protocol.

Raw reads of the genome were adapter-trimmed using Trimmomatic v.0.30, assembled using SPAdes and CDS were annotated through BV-BRC genome annotation service and RAST [34, 35]. Sequence was verified via BLAST (BLAST: Basic Local Alignment Search Tool) and PHASTEST servers [36]. Functions of CDS were confirmed through BLASTp (BLAST: Basic Local Alignment Search Tool) against non-redundant protein sequences (E < 10^− 5^). Presence of Antimicrobial resistance genes and virulence factors were annotated through CARD using the Resistance Gene Identifier (RGI) and VFDB [37] and were further analysed using the PARTRIC k-mer based AMR genes detection method [34]. Whole genome phylogenetic analysis of phage was analysed using the Virus Classification and Tree building online tool (VICTOR) [38]. Similar phage sequences were obtained from the NCBI nucleotide database and all pairwise comparisons of the sequences were analysed using phylogenomic inference and precise intergenomic distances, that were calculated using the Genome BLAST Distance Phylogeny approach (GBDP) with the algorithm ‘coverage’ and distance formulae *d*_*5*_, confidence levels were calculated using the recommended settings of GGDC (https://ggdc.dsmz.de/victor.php) [39]. An evolution tree with branch support was generated with FASTME 2.1.6.1 and SPR processing [40]. Branch support was inferred from 100 pseudo-bootstraps each, trees were visualised with PhyD3 using iTOL (*iTOL: Interactive Tree Of Life (embl.de)* and rooted at the midpoint [41]. VIRDIC (Virus Intergenomic Distance Calculator) was used to determine the phage intergenomic distance between its closest relatives via BLASTn and calculating the pairwise average nucleotide identity [42]. Visualization of the phage was produced using Proksee [43].

### Nucleotide sequence accession numbers

*S. maltophilia* strain (SM-BCU1) meta data can be found in the Sequence Read Archive (SRA) SUB14869520. The complete genome sequence of the phage was deposited in GenBank under the name *Stenotrophomonas* phage vB_SmaS_BCU1 and accession number PQ111865.1.

## Results

### Isolation and morphology

**Fig. 1:**
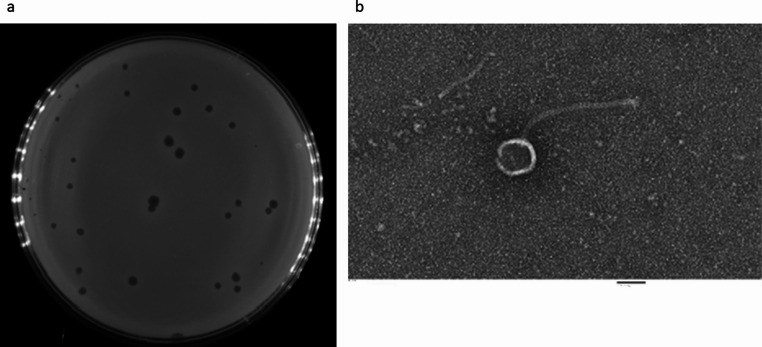
**(a**) Plaque formation by vB_SmaS_BCU1 in in a double-layer plaque assay. (**b**) Electron micrographs of bacteriophage. Magnification x250k fold magnification. Scale bar represents 50 nm

Phage vB_SmaS_BCU1 was isolated after propagation with its host. It produced clear plaques with sizes ranging between 1.0 mm and 2.0 mm (Fig. 1a). Transmission electron microscopy (Fig. 1b) indicated that this virus belongs to the Caudoviricetes family, and its morphology corresponds to the former family of *Siphoviridae*, with a long non-contractile tail. The phage has a head width of 60 *±* 5 nm, head length of 67 *±* 5 nm and its tail length was 213 *±* 10 nm.

### MOI, one-step growth and adsorption kinetics

MO1 0.01 achieved the highest phage titre with a value of 10^9^ PFU/mL.

**Fig. 2:**
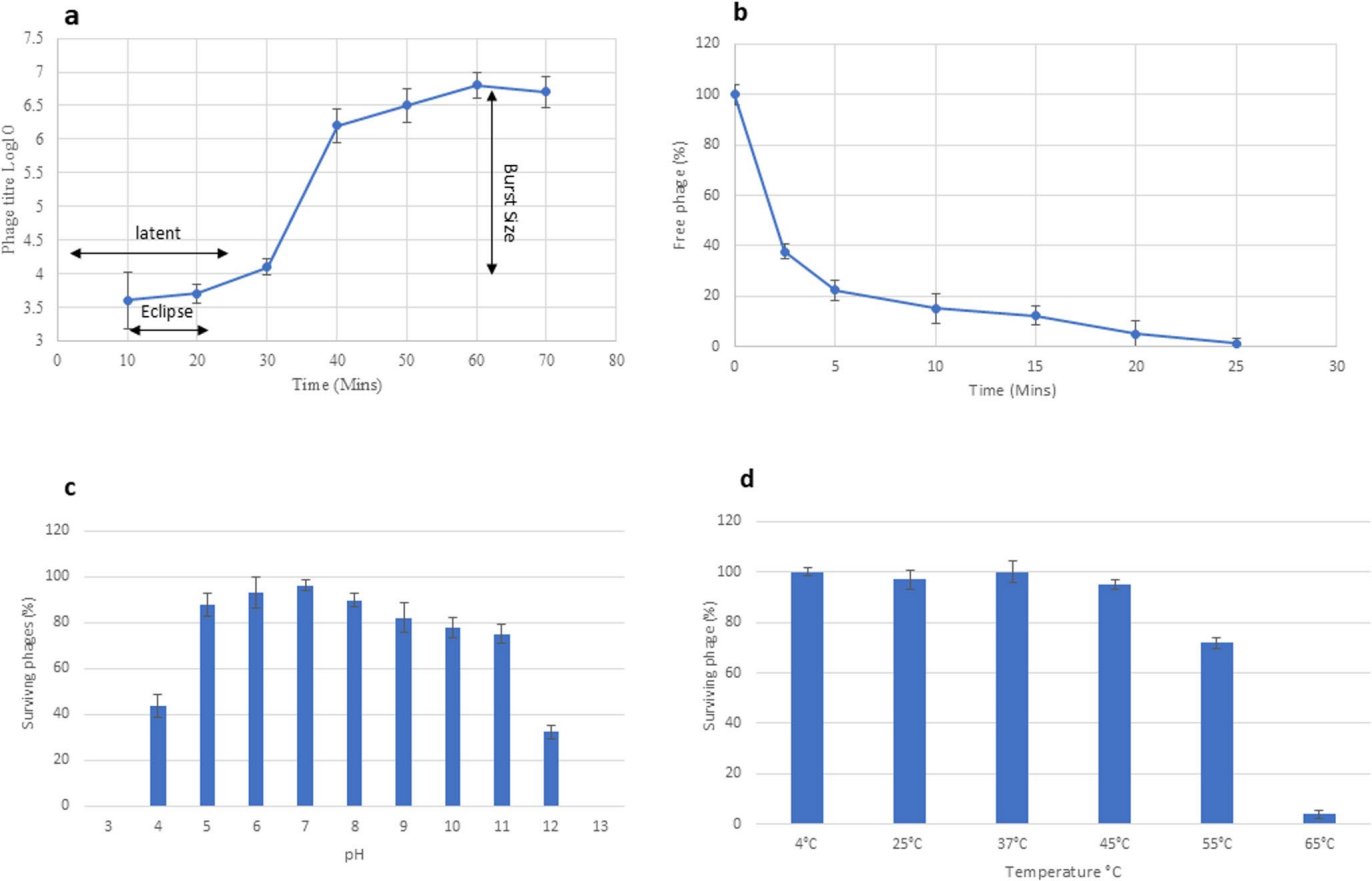
Biological properties of phage vB_Smas_BCU1. (**a**) One-step growth curve demonstrating triphasic growth pattern. (**b**) Phage adsorption with host. Time of exposure is represented by the X axis and Y axis is the percentage of free pages in solutions at specific time points. (**c**) Graph showing effects of various pH conditions on phages. Expressed as percentage of survived phages (**d**) Thermal stability of phages at different temperatures. Expressed as percentage of survived phages. Data obtained in all cases were from three independent experiments and represented as mean value +/- SD

Phage vB_SmaS_BCU1 had a latent period of 30 min and burst size was approximately 150 particles per bacterial cell (Fig. 2a). Regarding kinetics, vB_SmaS_BCU1 viral particles adsorbed over 88.9% to *S. maltophilia* strain SM-BCU1 within 10 min. Adsorption constant, *k* was determined as 1.9 × 10^− 8^ ml cell^− 1^ min^− 1^ (Fig. 2b).

####  Effect of pH and temperature on phage stability and host range

Phage vB_SmaS_BCU1 was stable at pH ranges of 4–12. Optimum pH with the highest percentage of survived phages, was pH 7, closely followed by pH 6. Survival of phage was dramatically reduced by 50% at pH ranges of 4 and 12. Activity of phage was completely inactivated at pH ranges of 3 and 13. Temperatures 4, 25, 37, 45, 55 and 65 °C were used to assess the thermal stability of the phage. Survival rates of phage were over 95% at temperatures ranging from 4 to 45 °C, rates decreased by 30% at 55 °C and by 90% at 65 °C (Fig. 2c & d).

The host range of the phage was assessed on 12 isolates, vB_SmaS_BCU1 could lyse 2 out of 3 *S. maltophilia* strains but no lysis was apparent against the other strains of bacteria tested.

### Genomic characterization of vB_SmaS_BCU1 genome

**Fig. 3:**
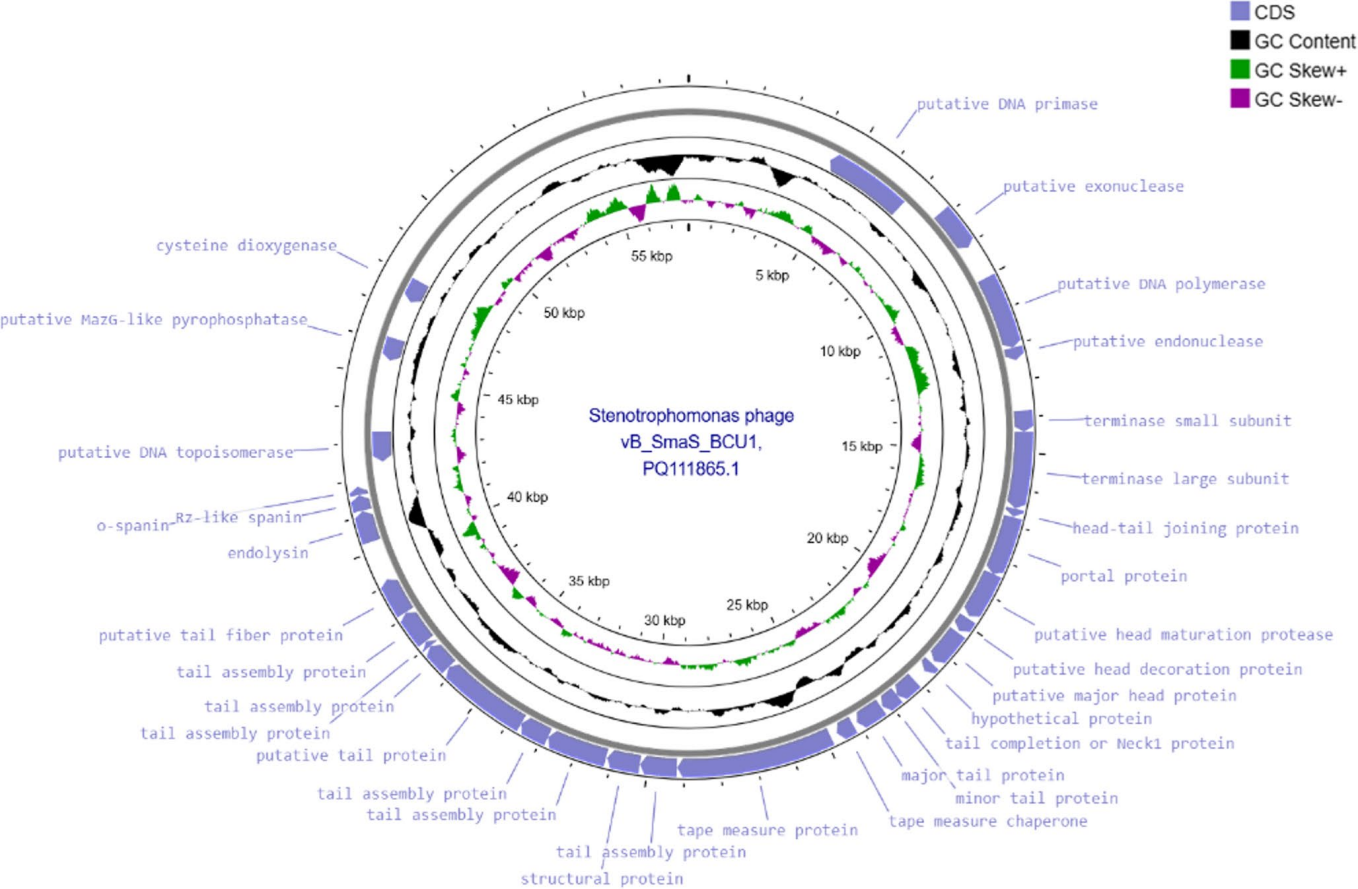
Circular presentation of phage vB_Smas_BCU1. Genomic annotation visualised through Proksee. Hypothetical proteins not shown

Whole genomic sequencing of vB_SmaS_BCU1 was undertaking using Illumina MiSeq platform. It is composed of linear ds DNA, with a length of 57,752 bp and GC Content 62.1%, No tRNA were detected using ARAGORN [44]. No virulence factor genes or antimicrobial resistant genes were found within the genome, analysis by PhageLead [45] revealed no genes related to temperate phages and RAST and BV-BRC [46] predicted 75 open reading frames (ORFs), 31 were annotated with known functions, whereas 44 were hypothetical proteins (Table S5) (Fig. 3 ).

Annotated proteins with known and similar functions were categorised into groups ORF 11 (DNA primase), ORF 14 (exonuclease), ORF 16 (DNA polymerase), ORF 17 (endonuclease) and ORF 47 (DNA topoisomerase) all play a role in DNA/RNA processing and metabolism [47, 48],. ORF 18 and 19 (terminase small and large subunits), are known to play vital roles in DNA packaging systems, the large subunit allows the ATP-powered translocation of DNA, whereas the small subunit can initiate the packaging of the genome [49]. The following ORFs were categorised as structural and assembly proteins. ORFs 20–24 and ORFs 27–40 are all associated with the structure of the neck and tail complex, commonly associated with siphophages and their facilitation of receptor binding [50]. Furthermore, ORFs 43–45 (endolysin, R_z_-like spanin and O-spanin) were classified as lysis proteins [51]. ORFs 53 and 56, (putative MazG-like pyrophosphatase and cysteine dioxygenase) had unknown functions.

#### Phylogenetic analysis

**Fig. 4:**
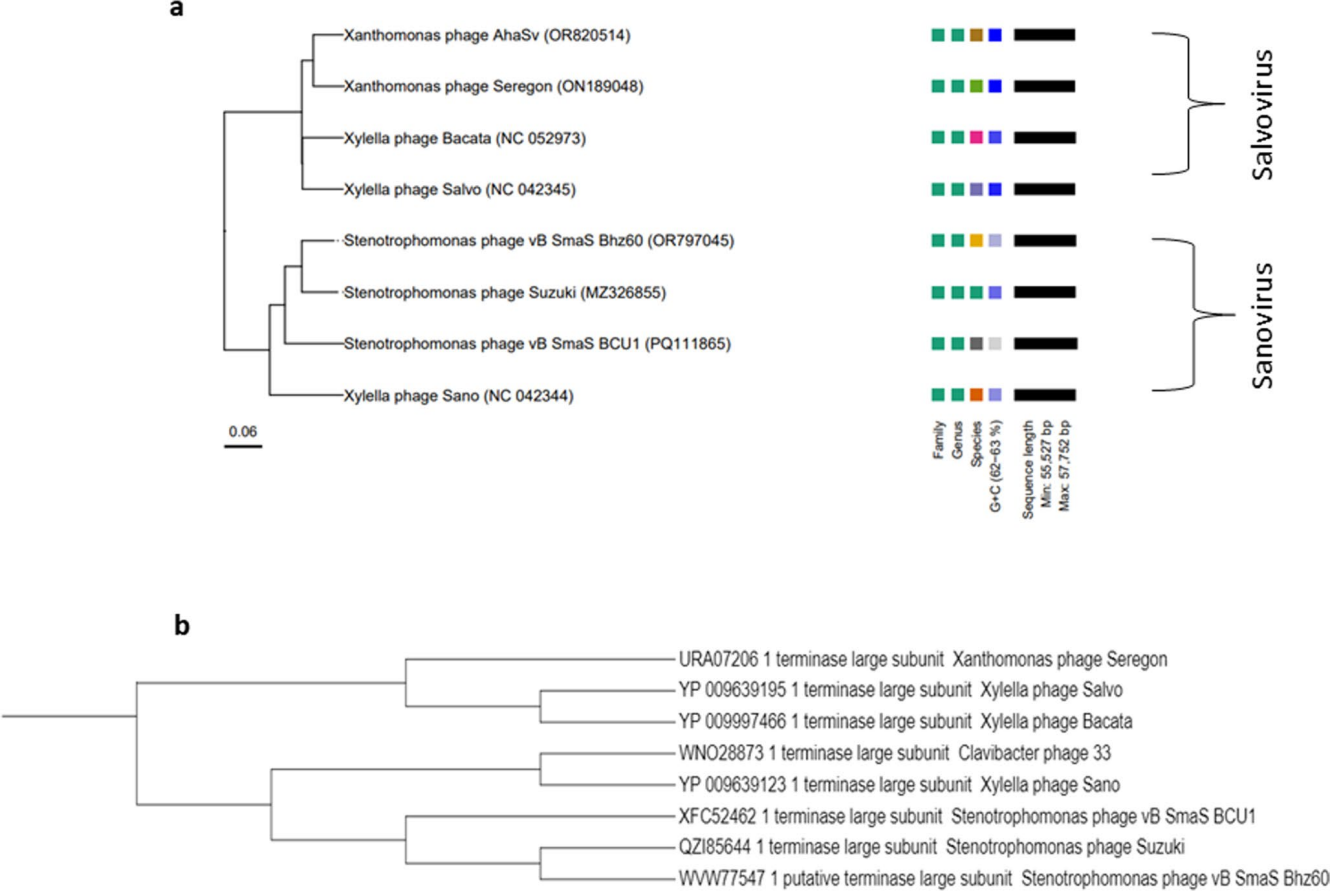
(**A**) Phylogenetic relatedness of 10 closely related strains to phage vB_SmaS BCU1. Based on whole genomic sequencing between hallmark and core genes via thresholds optimised to the ICTV classification using VICTOR (**B**) Viral conserved protein based phylogenetic tree illustrating evolutionary relatedness of the terminase large subunit of vB_SmaS_BCU1 and closely related phages

**Fig. 5:**
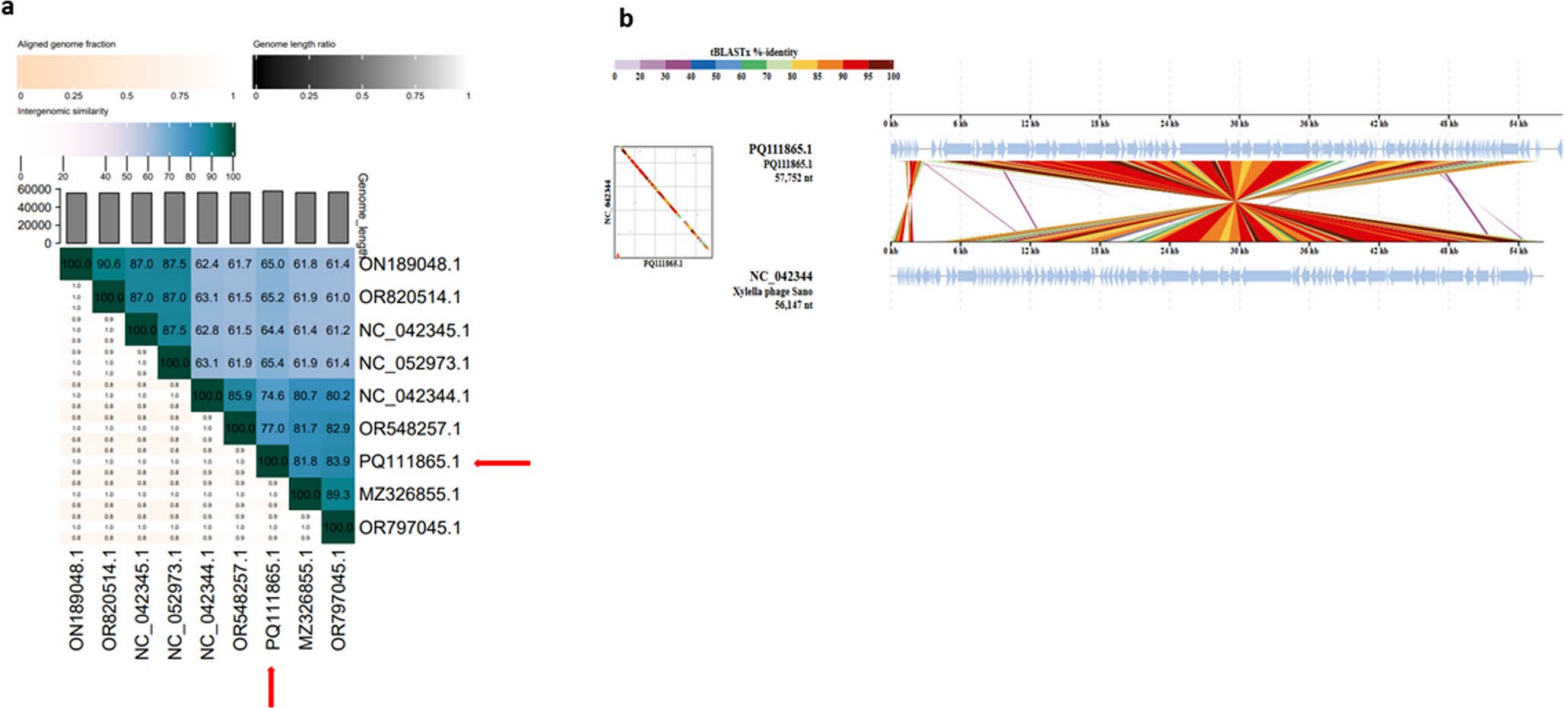
(**a**) Heatmap of comparative genome analysis of vB_SmaS BCU Phage using VIRDIC, indicating close homologs in BLASTn and estimating intergenomic similarities between the phages. The upper right half contains the intergenomic similarities between phage parings, with intensity of colour corresponding to level of similarity. Red arrows show the position of phage vB_SmaS-BCU-1 (**b**) Genomic alignment of phage vB_SmaS_BCU1 and phage Suzuki. Coloured blocks illustrate % identity of sequence calculated via tBLASTx using DiGAlign and visualised as a function implemented on ViPTree

BLASTn was used for comparative genomic analysis. eight of the closest phages were selected, with *Stenotrophomonas* phage Suzuki (MZ326855.1) demonstrating the highest similarity at 95.66% with a query coverage of 84%, phage Seregon (ON189048.1) had the lowest similarity at 78.62% with a query coverage of 67%. A phylogenetic tree using whole genomic sequences from the phages selected, was generated using VICTOR and visualised on iTOL (Fig. 4a). A heatmap based upon intergenomic similarities between vB_SmaS_BCU1 and its closest homologs in BLASTn was also generated using VIRIDIC. Results from the phylogenetic tree generated by VICTOR and heat map by VIRDIC (Fig. 5a), revealed phage vb_SmaS_BCU1 belonged to the family *Casjenviridae*, genus *Sanovirus*. It shared greater > 50% high level of nucleotide sequence similarity, with phages vB_SmaS_Bhz60 (OR797045.1), Suzuki (MZ326855.1) and Sano (NC_042344.1) and shared a linage with the genus Salvovirus. DiGAlign function from VipTree was used to visualise % identity of protein sequence with its closest homologs by comparing viral genome sequence similarities between phage Sano and BCU-1 using tBLASTx [52]. Phage vB_SmaS_BCU1 had greater than 5% nucleotide similarity to the phages stated, through BLASTn, suggesting phage BCU1 could be a separate genus within the subfamily [53]. This was further explored by assessing the evolutionary relationship between the closely related phages through a phylogenetic tree based upon the terminase large subunit (Fig. 4b). vB_SmaS_BCU1 is placed on a separate evolutionary branch but shares the same clade with phages vB_SmaS_Bhz60 and Suzuki.

#### Biofilm assay

**Fig. 6:**
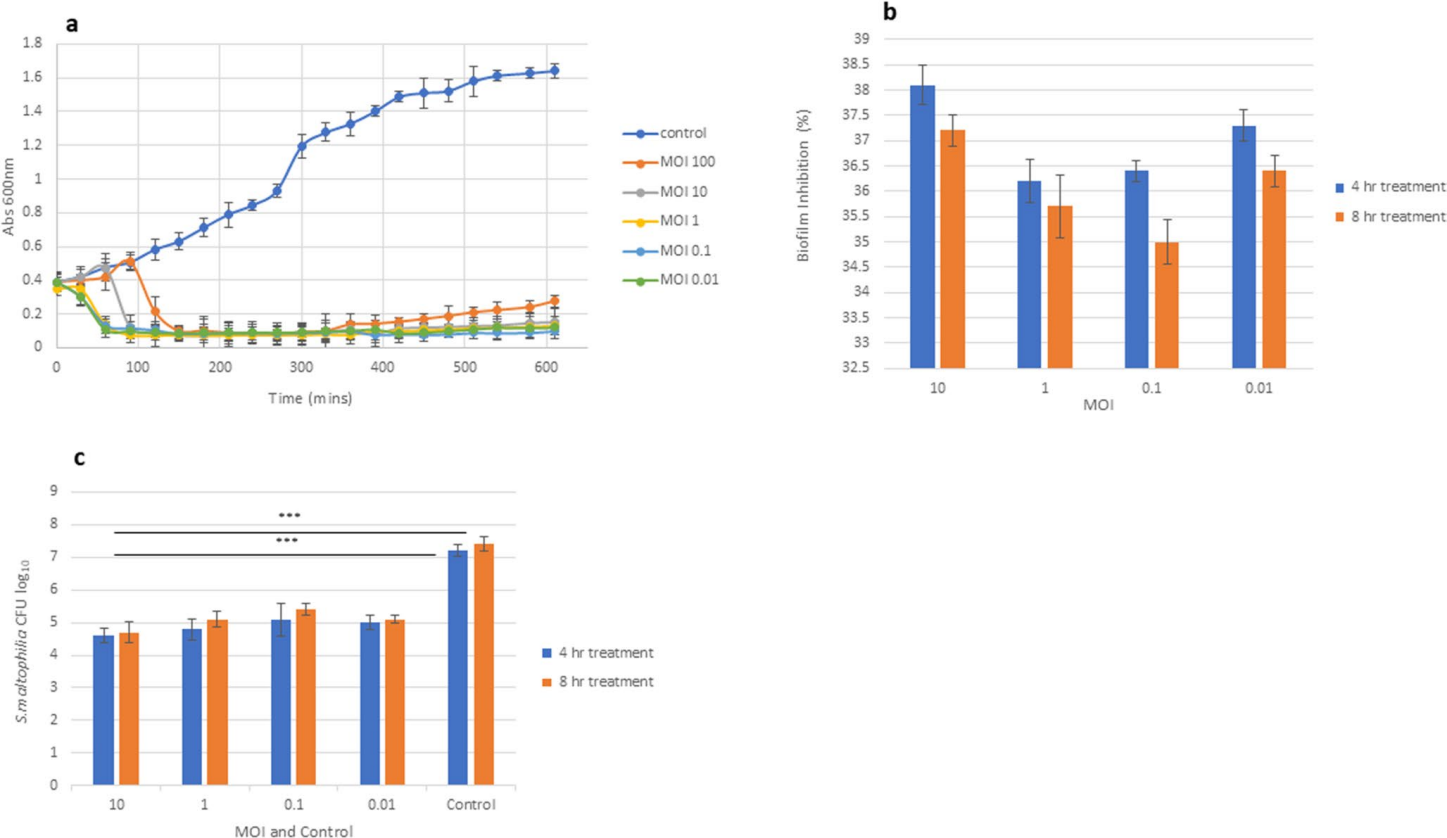
**(a**) Lysis kinetics of vB_SmaS BCU in vitro. *S. maltophilia* in exponential phase mixed with phage at different MOIs of 0.01, 0.1, 1, 10 & 100 and incubated at 30 °C for 10 h., data points taken every 30 min. Inhibition of bacteria evident from 60 min post infection. (**b**) Biofilm destruction of host bacteria using phages at MOI 0.01, 0.1, 1, 10 in a MBEC assay using resazurin, results expressed as percentage inhibited in relation to bacterial (**c**) experiments and represented as mean value +/- SD. ***P < 0.001, **P < 0.01 or *P < 0.05

Anti-Biofilm activity of the phage was determined via MBEC (Minimum Biofilm Eradication Concentration) assay system. Destruction of biofilm was determined via a resazurin assay measuring the metabolic activity of the cells after 4- and 8-hours post treatment. There was no statistical difference between the MOI’s evaluated and destruction of biofilm (Fig. 6b & c), however, MOI 10 demonstrated the highest biofilm destruction at 38.1% after 4-hours and 37.2% after 8-hours post treatment when compared to the control (untreated biofilm). MO1 0.1 and 1 had similar effects on the destruction of the biofilm after 4- and 8-hours post treatment hours (36.2%; 35.2% and 36.4%; 35.2%, respectively). MOI 0.01 illustrated the lowest percentage of biofilm destruction (35.8% and 35%).

The number of bacteria lysed by the phage within the MBEC assay were quantified via a CFU assay. All MOI’s demonstrated a statistical significance (^***^P < 0.001) when compared to the control (untreated biofilm), however MOI 10 had the largest reduction in bacterial density by 36.1% (2.6 log difference) and 36.4% (2.5 log difference) after 4-hours and 8-hours post treatment. The two lowest MOI’s, 0.1 and 0.01 had comparable results with an average of 32% reduction in bacterial load (2.1 log difference) after 4-hours, and 31% (2.1 log difference) after 8-hours post treatment, respectively.

#### Phage toxicity to fibroblasts

**Fig. 7:**
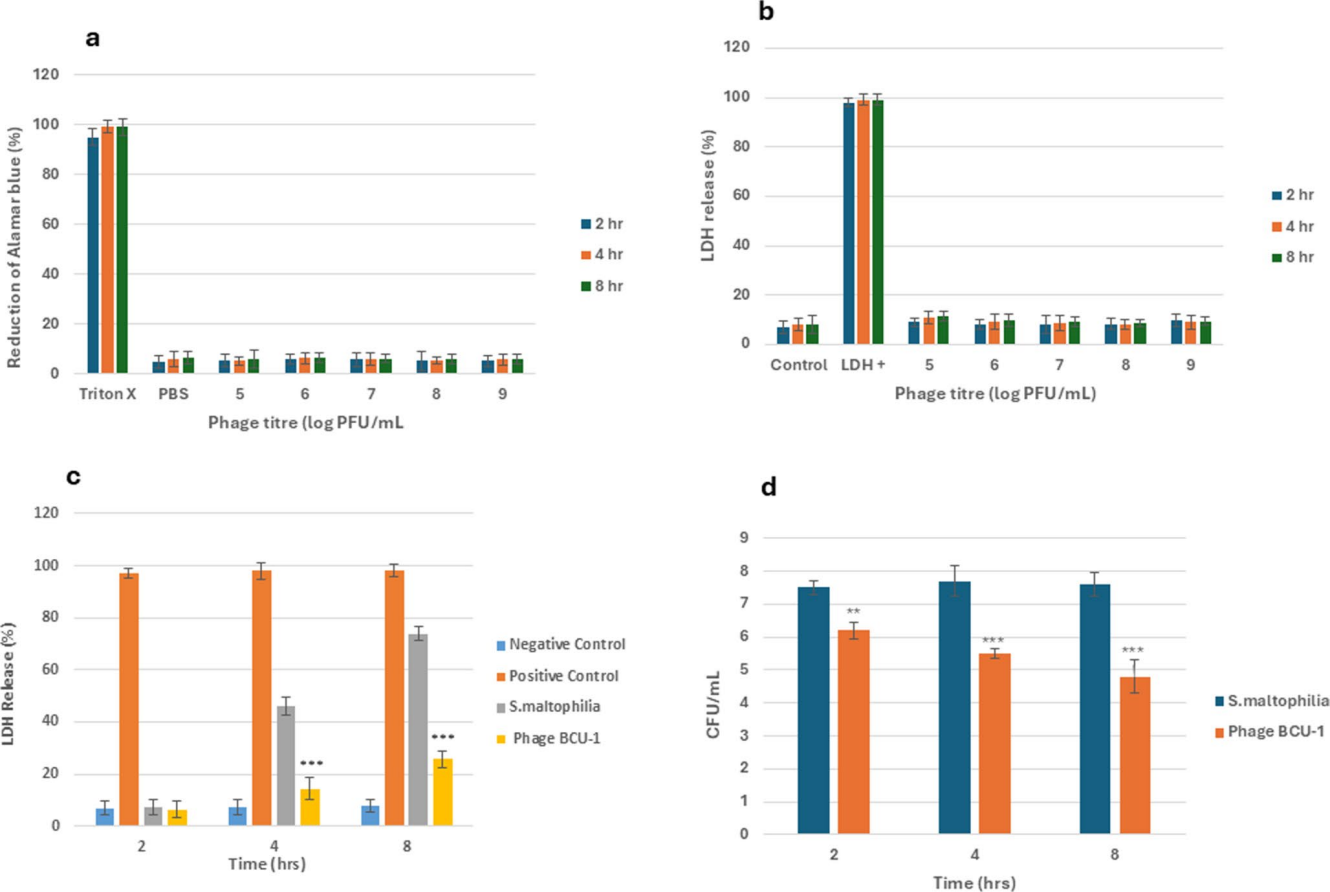
(**a**) Cell viability of fibroblasts treated with phage at different concentrations during 2-, 4- and 8-hour incubation, compared to triton-x treated positive control using AlamarBlue as cytotoxicity indicator, Results expressed as percentage reduction of resazurin. (**b**) LDH release assay from fibroblasts treated with phage at different concentrations during 2-, 4- and 8-hour incubation compared to LDH release positive control. (**c**) LDH release assay from fibroblasts infected with host bacteria treated with phage vB_Smas_BCU1 over 2-, 4- and 8-hours compared to untreated control. (**d**) CFU/mL of host bacteria treated with phage vB_Smas_BCU1 over 2-, 4- and 8-hours compared to untreated control. Data obtained in all cases were from three independent experiments and represented as mean value +/- SD. ^***^P < 0.001, ^**^P < 0.01 or ^*^P < 0.05

Various concentrations of the phage (10^5^ to 10^9^ PFU/mL) were used to determine the cytotoxicity effect on fibroblasts after 2-, 4- and 8-hours through an AlamarBlue assay. Results indicted, even at high concentrations, there was no detrimental effect on the fibroblasts. There was no statistical significance between the concentrations or the negative control. The LDH assay was used to determine any disruption within the cell membranes caused by the different phage concentrations, which could lead to cell death. Results (Fig. 7a and b) showed phage concentrations across all incubation times on the cell membranes produced similar amounts of LDH to the negative control and there was no statistical significance between them.

Fibroblast membrane integrity was also assessed through the LDH assay when host bacteria was inoculated onto the fibroblasts and challenged with the phage. Results indicated, after 2-hours, minimal amounts of LDH were released between the negative control, untreated cells (bacteria only) and treated cells (phage treated), however after 4-hours and 8-hours, phage vB_SmaS_BCU1 reduced the toxicity of bacterial infection towards fibroblast cells compared to the untreated cells by 68.9% after 4-hours and 65.2% after 8-hours, respectively. When compared to the positive LDH control, there was 85.4% and 73.7% difference after 4-hours and 8-hours, respectively. However, state of cell line did start to deteriorate over time, between 2- and 8-hours resulting in a 30% increase in LDH (Fig. 7c).

Phage activity against *S. maltophilia* strain SM-BCU1 on the surface of the cell line was determined by calculating the CFU/mL within all time frames tested, the phage was able to reduce the bacterial population compared to the control (untreated cells), after 2-hours there was a 12% (0.8) log reduction, 4 h a 23.1% (1.6 log) reduction and after 8 h a 31.4% (2.2 log) reduction, respectively (Fig. 7d).

.

## Discussion

*S. maltophilia* is an opportunistic pathogen with numerous intrinsic and extrinsic acquired resistant mechanisms, it is increasingly involved in tissue associated infections [54] The resistome of *S. maltophilia* and the emergence of high-level trimethoprim/sulfamethoxazole resistance among the genus *Stenotrophomonas* make it difficult treat [55]. Therefore, an alternative strategy to treat and control such infections is required.

In this study, a novel lytic phage was isolated and characterised using a clinical *S. maltophilia* strain SM-BCU1 responsible for a diabetic foot ulcer as the host organism. Through the double-layer agar assay, the phage produced transparent plaques, sizes ranging between 1.0 mm and 2.0 mm in size. Different in size could be attributed to T-even lysis inhibition phenomenon, where larger phage virions can cause smaller plaque sizes [56]. TEM morphology of phage particles suggested it was a siphophage, classification and genus were confirmed through genomic analysis, placing the phage in the family *Casjensviridae*, genus Sanovirus.

A key indicator of phage lysis proficiency is through the MOI, lower the MOI result in fewer phage particles required to lyse the same number of bacteria [57]. Phage vB_SmaS_BCU1 had an optimal MOI of 0.01, similar to other *S. maltophilia* phages, vB_SmaS_QH3 PP932004.1 [58] and phage BUCT603 [59] suggesting, the highest number of progenies are produced at this MOI.

Adsorption is crucial in phage infection and within the context of phage therapy, understanding the process is essential [60]. In this study, adsorption rate *k* (ml cells^− 1^ min^− 1^) for vB_SmaS_BCU1 was 1.9 × 10^− 8^ ml cell^− 1^ min^− 1^, approximately 89% of the phage had adsorbed into the host within 10 min, demonstrating a fast adsorption. Other Stenotrophomonas phages, BCUT 609 [59], BCUT 555 [61] and Ps15 [62] demonstrated > 90% adsorption within 10 min or less, whereas phage CUB19 took over 20 min to adsorb 90% into the host bacterium with an adsorption rate of 1.59 × 10^− 9^ ml cell^− 1^ min^− 1^ [63]. Differences in adsorption can be attributed to phage type, phage receptor specificity, accessibility, tail structure and binding efficacy [64]. Notably, a high adsorption rate is desirable, which leads to rapid infection and bacterial eradication.

Another crucial element within phage therapy is its biocontrol application, phage latent period and burst size are essential parameters within this paradigm. Phage vB_SmaS_BCU1 had a latent period of 30 min and burst size of 150 PFU/cell, higher than Sanovirus phage Sano (100 *±* 10.1 PFU per cell) [65], less than phage BUCT 555 (30 min latent period and a burst size of 204 PFU per cell) and similar to Stenotrophomonas phage CUB19 (155 PFU per cell). Stability of phage BCU-1 in environmental conditions demonstrated its integrity was maintained in a range of temperatures and pH values, suggesting its stability is ideal for antimicrobial drug formulation and production [66]. Host range of phage vB_SmaS_BCU1 can be regarded as narrow, due to the limited strains of *S. maltophilia* evaluated and seems to be specific to *Stenotrophomonas* as none of the closely related strains were lysed by the phage. More strains will be needed to fully evaluate its host range.

Bacteriolytic activity of a phage is a crucial step in evaluating lysis activity, in this regard in vitro assays were undertaken. BCU-1 was able to lyse the clinical strain at the MOI’s tested over a 10-hour period, with significant decrease in the bacterial population less than 100 min post infection for MOI’s 0.01–10.Whereas inhibitory activity of MOI 100, started to decrease at around 5 h, (Fig. 6a), this could be due to greater selective pressure and the emergence a phage resistant population [67]. MOI is a critical parameter when characterising a phage for possible phage therapy, a reason for the similar outcomes between MOIs 0.01–10 within this assay could be due to phage-adsorption kinetics, burst size & replication cycles (including lysis timing), cell saturation and phage competition which all can be compounded by experimental conditions [68], yet the findings do indicate vB_SmaS_BCU1 can effectively lyse bacteria at low MOIs with results comparable to higher MOIs within the 10 h time frame.

Chronic wounds and biofilm related infections especially in diabetic foot ulcers can be difficult to treat due to the multifactorial pathophysiological elements attributed to them and are detrimental to health [69]. Biofilms are known to contain extracellular polysaccharides (EPS), a cellular matrix enriched with eDNA (extracellular DNA) and amyloids that limit the effect of antibiotics and contribute to the resistance of the biofilm [70], notably, it has been reported up to 98% of *S. maltophilia* clinical isolates are known to form biofilms on host tissues and abiotic substances [71]. In this context antibiofilm activity of vB_SmaS_BCU1 was evaluated against preformed SM-BCU1 24-hour biofilm using different MOIs in a MBEC assay. MOI 10 exhibited the greatest biofilm disruption (Fig. 6b). Moreover, all MOIs demonstrated a statistically significant reduction (P< ***0.001) of the biofilm compared to the control with no significant difference between the MOIs (Fig. 6c). The similarity in results may be attributed to biofilm defences, such as adsorption traps, diffusion inhibition, (phage cannot reach the denser cells due to the extracellular matrix) and when it does, phage proliferation is inhibited due to metabolically less active cells, moreover, phage resistant bacteria are known to shield phage sensitive bacteria in a process known as the wall effect, reducing phage predation [72]. Notably, environmental mutations within spatial architecture of the biofilm can produce phage resistant cells and alter phage receptors [72–74]. Regardless, across all MOI’s, phage treatment reduced the biofilm by approximately 36% (2.2 log reduction) after 4-hour post treatment and 35% (2.5 log reduction) after 8-hours post treatment, suggesting the destruction of the biofilm is phage mediated. Further investigations are required to assess phage activity against more mature biofilms, the synergistic effects of phage-antibiotic combinations and spatial analysis of the biofilms at different time intervals.

Phage vB_SmaS_BCU1 has a standard lysis mechanism, organised as lysis cassettes, however a protein coding for holin was not found, yet lysis and destruction of the bacterial membrane was evident within the lysis and antibiofilm assays (Fig. 6b & c) suggesting host cell lysis is occurring with the endolysin (associated with non-annotated holin and anti-holin protein factors) causing inner membrane-peptidoglycan disruption while the spanins cause outer membrane disruption [75]. Moreover, high sequence similarity of the endolysin gene was found in Xanthomonas phage AhaSv (OR820514) with 82.9% homology, query cover 94% and Xylella phage Salvo (NC_042345) with an 82.18% homology and 94% query cover. Similarly, the spanin genes were also found to be of high similarity in these Saloviruses, there is an overlap with phages belonging to Sanovirus genus but at lower homology, for example phage Suzuki (MZ326855.1) with 36.4% homology and 97% query cover.

Genomic annotation of phage vB_SmaS_BCU1 indicated it possesses all the basic DNA replication and packaging units, genes encoding structural and lysis proteins, including additional functional proteins such as cysteine dioxygenase, which has been speculated to be involved in evasion of host receptors and play a role in DNA packaging [76], whereas, MazG-like pyrophosphatase could be involved as an antagonist against the Bacterial Cyclic oligonucleotide-Based Anti-phage Signalling System (CBASS) resulting in invasion and phage propagation within the host [77, 78]. No antimicrobial resistant genes, toxin-related genes were discovered, moreover, PHATEST [36] detected no prophage related genes or virulence genes within the genome. BLASTn and VICTOR analysis of vB_SmaS_BCU1, revealed high sequence similarity with closely related phages Suzuki, Bhz60, and Sano (Fig. 4a). Furthermore, the same phages were clustered together in an evolutionary tree using the terminase large subunit (Fig. 4b). Highest genomic similarity through VIRDIC (Fig. 5a), placed phage Suzki (MZ2326855.1) as the closest relative to phage BCU-1 with a score of 89.3. Genomic alignment through DiGAlign showed protein coding genes of vB_SmaS_BCU1 and Suzuki shared an identity, averaging 60–100% (Fig. 5b). Contextualizing the genomic phylogenetic information obtained, a case can be made for phage BCU-1 to be considered a novel distant subspecies of the genus *Sanovirus* Under ICTV recommendations [79, 80].

One of the simplest and safest ways to treat skin or tissue infections is with topical medication, phages are different to antibiotics, they can self-replicate and maintain high concentrations within the body [81], but also interact with eukaryotic cells, therefore it was important to assess the cytotoxicity of vB_SmaS_BCU1. This study found even at high phage lysate concentrations; there was no negative impact on the cell line (Fig. 7a & b) and lysis of bacteria by the phage did not completely deteriorate the cell line through inflammation and apoptosis after 8-hours (Fig. 7c & d). These results share similarities with other studies [32, 33], however notable differences suggest, different cell types can influence phage uptake, and the type of phage and or size can affect interaction with mammalian cells [82]. Additional factors including pharmacokinetics, pharmacodynamics, and phage inactivation warrant further investigation, and further studies are required to examine phage activity against other *S. maltophilia* wound strains and cell lines, as well as elucidating these processes within a wound model. Nevertheless, results suggest phage vB_SmaS_BCU1 is not toxic to human fibroblasts, can effectively inhibit the infectious effects of the host strain and to our knowledge the first direct evidence of *S. maltophilia* specific phage activity on human dermal fibroblasts.

## Conclusion

In this study, a new member of the *Casjenviridae* was isolated and characterised through biological and genomic analysis. *Stenotrophomonas* phage vB_SmaS_BCU-1 is a dsDNA virus, with no integrase, antibiotic resistant or toxic genes. The phage demonstrated a lytic nature and antibiofilm activity with efficient kinetics, and deemed safe, when used in a human cell model. A primary limitation of this study is the narrow host range; future studies will focus on expanding host range and its synergist potential with antibiotics.

## Supplementary Information

Below is the link to the electronic supplementary material.

Supplementary Material 1 (DOCX 98.2 KB)

## Data Availability

All data generated or analysed during this study are included in this published article (and supplementary information files). *S. maltophilia* strain (SM-BCU1) meta data can be found in the Sequence Read Archive (SRA) SUB14869520. Phage vB_SmaS_BCU1 Accession number is [PQ111865.1](https://www.ncbi.nlm.nih.gov) and has been deposited into Genbank.
