# Supplementary material for: Isolation and characterisation of a novel Stenotrophomonas maltophilia phage vB_SmaS_BCU-1 with evaluation of mammalian cell safety
Source: Eur J Clin Microbiol Infect Dis. 2026 Jan 30;45(5):1333–48. doi: 10.1007/s10096-025-05395-z (PMC13222311; doi:10.1007/s10096-025-05395-z)
Supplement: Supplementary file 1 — Supplementary Material 1 (DOCX 98.2 KB) [file 10096_2025_5395_MOESM1_ESM.docx]

**Genomic annotation of strain SM-BCU1 *(S.maltophilia*)**

**Table S1: Protein features of SM-BCU1**

| Protein Features | Number |
| --- | --- |
| Hypothetical proteins | 1877 |
| Proteins with functional assignments | 5843 |
| Proteins with EC Number assignments | 1601 |
| Proteins with GO assignments | 1400 |
| Proteins with Pathway assignments | 1238 |
| Proteins with PATRIC genus-specific family (PLfam) assignments | 964 |
| Proteins with PATRIC cross-genus family (PGfam) assignments | 7511 |

The annotation was done in Bv-BRC using the Comprehensive Genome Analysis tool. It included 1,877 hypothetical proteins and 5,843 proteins with functional assignments The proteins with functional assignments included 1,601 proteins with Enzyme Commission (EC) numbers, 1,400 with Gene Ontology (GO) assignments, and 1,238 proteins that were mapped to KEGG pathway^.^ PATRIC annotation included two types of protein families, and this genome has 964 proteins that belong to the genus-specific protein families (PLFams) and 7,511 proteins that belong to the cross-genus protein families (PGFams) ([Bacterial and Viral Bioinformatics Resource Center | BV-BRC](https://www.bv-brc.org/))

**Table S2: Specialty Genes**

|  | Source | Genes |
| --- | --- | --- |
| Antibiotic Resistance | CARD | 5 |
| Antibiotic Resistance | NDARO | 1 |
| Antibiotic Resistance | PATRIC | 65 |
| Drug Target | DrugBank | 12 |
| Drug Target | TTD | 1 |
| Transporter | TCDB | 29 |
| Virulence Factor | PATRIC_VF | 3 |
| Virulence Factor | VFDB | 28 |
| Virulence Factor | Victors | 22 |

Many of the genes annotated have homology to known transporters virulence factors^[^drug targets and antibiotic resistance genes. The number of genes and the specific source database where homology was found is provided ([Bacterial and Viral Bioinformatics Resource Center | BV-BRC](https://www.bv-brc.org/))

**Table S3: Antimicrobial Resistance genes**

| AMR Mechanism | Genes |
| --- | --- |
| Antibiotic activation enzyme | KatG |
| Antibiotic inactivation enzyme | APH(3')-II/APH(3')-XV |
| Antibiotic target in susceptible species | Alr, Ddl, dxr, EF-G, EF-Tu, folA, Dfr, folP, gyrA, gyrB, Iso-tRNA, kasA, MurA, rho, rpoB, rpoC, S10p, S12p |
| Antibiotic target protection protein | BcrC |
| Antibiotic target replacement protein | fabV |
| Efflux pump conferring antibiotic resistance | EmrAB-OMF, EmrAB-TolC, MacA, MacB, MdtABC-OMF, MdtABC-TolC, MexXY-OMP |
| Gene conferring resistance via absence | gidB |
| Protein altering cell wall charge conferring resistance | GdpD, PgsA |
| Protein modulating permeability | OprB family, OprD family |
| Regular modulating expression of antibiotic resistance genes | H-NS, OxyR |

The Genome Annotation Service in PATRIC uses k-mer-based AMR genes detection method, which utilizes PATRIC’s curated collection of representative AMR gene sequence variants and assigns to each AMR gene functional annotation, broad mechanism of antibiotic resistance, drug class and, in some cases, specific antibiotic it confers resistance to

**Figure S1; Phylogenetic relatedness of closely related strains to *S.maltophilia* SM-BCU-1 based on whole genomic sequencing between hallmark and core genes via thresholds optimised by using Type (Strain) Genome Server (TYGS) (**[**https://tygs.dsmz.de**](https://tygs.dsmz.de)**)**


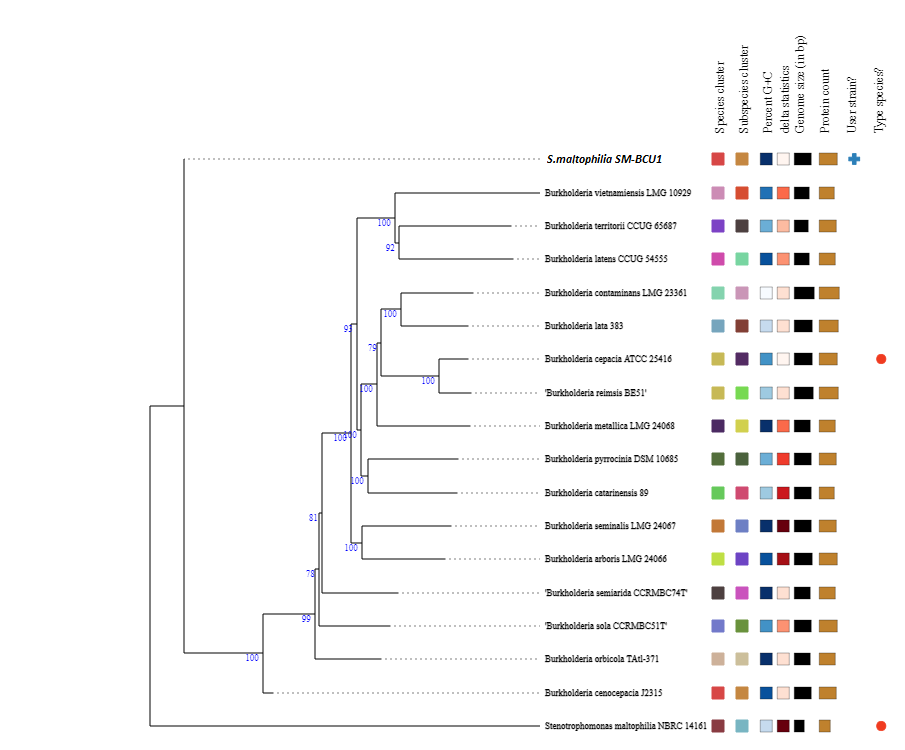


**Table S4 :Antibiotic biogram of SM-BCU1**

| **Antibiotic** | SXT | LVX | CIP | MIN | AMP | GEN | PIP/TAZ | ERY | MER |
| --- | --- | --- | --- | --- | --- | --- | --- | --- | --- |
| **Strain** |  |  |  |  |  |  |  |  |  |
| SM-BCU1 | S | S | I | S | R | R | I | R | I |

SXT - trimethoprim / sulfamethoxazole; LVX – levofloxacin; CIP – Ciprofloxacin; MIN – Minocycline; AMP – Ampicillin; GEN – Gentamicin; PIP/TAZ – Piperacillin/Tazobactum; ERY – Erythromycin; MER – Meropenem

R – Resistant; S – Sensitive; I- Intermediate

**Fig S2: Formulae used to calculate inhibition of Biofilm**

$$x=\frac{\left( E_{ox} \right)\lambda_{2}A\lambda_{1^{-}}\left( E_{0x} \right)\lambda_{1}A\lambda_{2}}{\left( E_{red} \right)\lambda_{2}A{'\lambda}_{1^{-}}\left( E_{red} \right)\lambda_{1}A'\lambda_{2}}x100$$

*E_o_*_x_ - molar extinction coefficient alamar blue oxidized (blue)

*E_red_* - molar extinction coefficient alamar blue reduced form (pink)

*A* - Absorbance of test wells

*A*$'$- Average Absorbance of Positive control well

λ_1_ – 570nm

λ_2_ – 600nm

**Fig S3: Formulae used to quantify bacteria within biofilm**

*Log_10_ (CFU/mm^2^) = Log_10_[(X/B)(V/A)/D+1]*

*X – CFU*

*B – Volume plated (0.01ml)*

*V – Volume of well (.2ml)*

*A – Peg surface area (46.63mm^2^)*

*D - Dilution*

Log_10_ reduction was calculated by using the formulae:

*Mean Log_10_ untreated Control pegs - Mean Log_10_ Treated pegs*

**Table S5: Annotation of open reading frames : *Stenotrophomonas* phage vB_SmaS_BCU1 and accession number** [**PQ111865.1**](https://www.ncbi.nlm.nih.gov/nuccore/PQ111865.1)

| **ORF** | **Start** | **End** | **Strand** | **Predicited function** |
| --- | --- | --- | --- | --- |
| 1 | 47 | 739 | Positve | hypothetical protein |
| 2 | 741 | 1151 | Positve | hypothetical protein |
| 3 | 1148 | 1327 | Positve | hypothetical protein |
| 4 | 1327 | 1467 | Positve | hypothetical protein |
| 5 | 1473 | 1664 | Positve | hypothetical protein |
| 6 | 1661 | 1852 | Positve | hypothetical protein |
| 7 | 1932 | 2126 | Positve | hypothetical protein |
| 8 | 2130 | 2411 | Positve | hypothetical protein |
| 9 | 3490 | 3969 | Positve | hypothetical protein |
| 10 | 4064 | 4366 | Negative | hypothetical protein |
| 11 | 4404 | 6911 | Negative | putative DNA primase |
| 12 | 6915 | 7181 | Negative | hypothetical protein |
| 13 | 7336 | 7779 | Positve | hypothetical protein |
| 14 | 7839 | 9071 | Positve | putative exonuclease |
| 15 | 9154 | 9954 | Positve | hypothetical protein |
| 16 | 10028 | 12088 | Positve | putative DNA polymerase |
| 17 | 12085 | 12441 | Positve | putative endonuclease |
| 18 | 13834 | 14424 | Positve | terminase small subunit |
| 19 | 14424 | 16529 | Positve | terminase large subunit |
| 20 | 16536 | 16763 | Positve | head-tail joining protein |
| 21 | 16777 | 18435 | Positve | portal protein |
| 22 | 18428 | 19765 | Positve | putative head maturation protease |
| 23 | 19830 | 20252 | Positve | putative head decoration protein |
| 24 | 20308 | 21330 | Positve | putative major head protein |
| 25 | 21412 | 21708 | Positve | hypothetical protein |
| 26 | 21736 | 22083 | Positve | hypothetical protein |
| 27 | 22080 | 22715 | Positve | Neck1 protein |
| 28 | 22708 | 23220 | Positve | minor tail protein |
| 29 | 23251 | 24039 | Positve | major tail protein |
| 30 | 24162 | 24668 | Positve | tape measure chaperone |
| 31 | 24857 | 29194 | Positve | tape measure protein |
| 32 | 29205 | 30218 | Positve | tail assembly protein |
| 33 | 30218 | 31138 | Positve | structural protein |
| 34 | 31161 | 32852 | Positve | tail assembly protein |
| 35 | 32854 | 33675 | Positve | tail assembly protein |
| 36 | 33675 | 36202 | Positve | putative tail protein |
| 37 | 36203 | 36967 | Positve | tail assembly protein |
| 38 | 36970 | 37140 | Positve | tail assembly protein |
| 39 | 37150 | 38112 | Positve | tail assembly protein |
| 40 | 38116 | 39162 | Positve | putative tail fiber protein |
| 41 | 39174 | 39908 | Positve | hypothetical protein |
| 42 | 39877 | 40275 | Positve | hypothetical protein |
| 43 | 40272 | 41150 | Positve | endolysin |
| 44 | 41161 | 41598 | Positve | Rz-like spanin |
| 45 | 41600 | 41833 | Positve | o-spanin |
| 46 | 42201 | 42452 | Negative | hypothetical protein |
| 47 | 42456 | 43334 | Negative | putative DNA topoisomerase |
| 48 | 43347 | 43463 | Negative | hypothetical protein |
| 49 | 43463 | 44200 | Negative | hypothetical protein |
| 50 | 44193 | 44636 | Negative | hypothetical protein |
| 51 | 44645 | 45223 | Negative | hypothetical protein |
| 52 | 45213 | 45488 | Negative | hypothetical protein |
| 53 | 45485 | 46165 | Negative | putative MazG-like pyrophosphatase |
| 54 | 46175 | 46675 | Negative | hypothetical protein |
| 55 | 46759 | 47211 | Negative | hypothetical protein |
| 56 | 47342 | 48019 | Negative | cysteine dioxygenase |
| 57 | 48049 | 48393 | Negative | hypothetical protein |
| 58 | 48572 | 48778 | Negative | hypothetical protein |
| 59 | 48771 | 49085 | Negative | hypothetical protein |
| 60 | 49085 | 49243 | Negative | hypothetical protein |
| 61 | 49248 | 49829 | Negative | hypothetical protein |
| 62 | 49826 | 50320 | Negative | hypothetical protein |
| 63 | 50333 | 50506 | Negative | hypothetical protein |
| 64 | 50508 | 50753 | Negative | hypothetical protein |
| 65 | 50755 | 51045 | Negative | hypothetical protein |
| 66 | 51042 | 51371 | Negative | hypothetical protein |
| 67 | 51368 | 51544 | Negative | hypothetical protein |
| 68 | 51541 | 52020 | Negative | hypothetical protein |
| 69 | 52017 | 52247 | Negative | hypothetical protein |
| 70 | 52244 | 53995 | Negative | hypothetical protein |
| 71 | 54017 | 54511 | Negative | hypothetical protein |
| 72 | 54517 | 54930 | Negative | hypothetical protein |
| 73 | 55025 | 55159 | Positve | hypothetical protein |
| 74 | 55479 | 55673 | Positve | hypothetical protein |
| 75 | 55683 | 55832 | Positve | hypothetical protein |
